# Proprotein convertases in high-density lipoprotein metabolism

**DOI:** 10.1186/2050-7771-1-27

**Published:** 2013-09-18

**Authors:** Seungbum Choi, Ron Korstanje

**Affiliations:** 1 The Jackson Laboratory, Bar Harbor, ME, USA; 2 Graduate School of Biomedical Sciences, University of Maine, Orono ME, USA

**Keywords:** Proprotein convertase subtilisin/kexin, High-density lipoprotein cholesterol, Reverse cholesterol transport

## Abstract

The proprotein convertase subtilisin/kexins (PCSKs) are a serine endopeptidase family. PCSK members cleave amino acid residues and modulate the activity of precursor proteins. Evidence from patients and animal models carrying genetic alterations in PCSK members show that PCSK members are involved in various metabolic processes. These studies further revealed the molecular mechanism by which genetic alteration of some PCSK members impairs normal molecular and physiological functions, which in turn lead to cardiovascular disease. High-density lipoprotein (HDL) is anti-atherogenic as it removes excessive amount of cholesterol from blood and peripheral tissues. Several PCSK members are involved in HDL metabolism. PCSK3, PCSK5, and PCSK6 process two triglyceride lipase family members, endothelial lipase and lipoprotein lipase, which are important for HDL remodeling. Recent studies in our lab found evidence that PCSK1 and PCSK9 are also involved in HDL metabolism. A mouse model carrying an amino acid substitution in PCSK1 showed an increase in serum apolipoprotein A1 (APOA1) level. Another mouse model lacking PCSK9 showed a decrease in APOE-containing HDL. In this review, we summarize the role of the five PCSK members in lipid, glucose, and bile acid (BA) metabolism, each of which can influence HDL metabolism. We propose an integrative model in which PCSK members regulate HDL metabolism through various molecular mechanisms and metabolic processes and genetic variation in some PCSK members may affect the efficiency of reverse cholesterol transport. PCSK members are considered as attractive therapeutic targets. A greater understanding of the molecular and physiological functions of PCSK members will improve therapeutic strategies and drug efficacy for cardiovascular disease where PCSK members play critical role, with fewer adverse effects.

## Introduction

In the past two decades nine members of the highly conserved bacterial subtilisin- and yeast kexin-like serine protease family have been discovered and characterized. Analyses in animal models and in patients with mutations in PCSK members have revealed the molecular mechanism by which genetic alterations in PCSK members affect molecular and physiological functions in various metabolic diseases and endocrinological defects [1-4]. However, our understanding of how PCSK members are involved in HDL metabolism is still limited. The HDL biology has been updated in many reviews during the past decades [5-10], and we briefly summarize several key points: The major function of HDL is to remove excessive amounts of cholesterol from peripheral tissues via a mechanism called reverse cholesterol transport (RCT). HDL takes up cholesterol from low-density lipoprotein (LDL) or macrophage foam cells in atherosclerotic lesions and then delivers it to the liver for recycling or excretion. As a high level of HDL correlates with low risk of atherosclerotic cardiovascular diseases, HDL is considered an anti-atherogenic factor. Thus, abnormal regulation of HDL synthesis, remodeling and catabolism causes reduced HDL cholesterol concentration and HDL function, which then promotes the risk or progression of the disease.

Some PCSK members were previously found to be important in HDL metabolism. Data from our lab shows that PCSK1 is involved in regulating the levels of serum apolipoprotein A1 (APOA1), which is the major protein for HDL formation and remodeling (Choi et al., in preparation). PCSK3, PCSK5 and PCSK6 process two members in the triglyceride (TG) lipase family: endothelial lipases (EL) and lipoprotein lipases (LPL) [11] that play important role in regulating the HDL cholesterol concentration. We have also shown that PCSK9 influences HDL cholesterol concentration by regulating APOE-containing HDL levels [12].

### Protein structure and principle of PCSK

**Figure 1:**
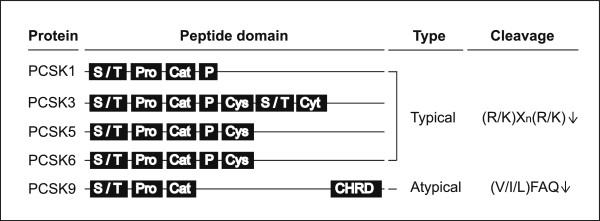
**Schematic illustration for the protein structure; typical and atypical cleavage activies of human PCSK1, PCSK3, PCSK5, PCSK6, and PCSK9.** The figure illustrates the peptide domains and cleavage site of human PCSK1, PCSK3, PCSK5, PCSK6, and PCSK9. The five PCSK members consist of several peptide domains: a signal peptide/transmembrane domain (S/T) at the amino-terminus, a pro-segment domain (Pro), a catalytic domain (Cat), a P domain (P), a Cys-rich domain (Cys), a cytoplasmic tail (Cyt), and a Cys-His-rich domain (CHRD) at the carboxyl-terminus. Three protein domains are common between five PCSK members: S/T, Pro, and Cat domains. Typical members (PCSK1, PCSK3, PCSK5, and PCSK6) cleaves single or paired basic amino acids with the motif (R/K)X_*n*_(R/K)↓, where the arrow indicates the cleavage site and X_*n*_ corresponds to a 0-, 2-, 4-, or 6-amino-acid spacer at (R/K)X_*n*_(R/K)↓, meanwhile atypical member (PCSK9) cleaves non-basic residues at the C-terminal end of the motif (V/I/L)FAQ↓.

PCSK proteins consist of several peptide domains: a signal peptide/transmembrane domain at the amino-terminus, a prosegment domain, a catalytic domain, a P domain, a Cys-rich domain, a cytoplasmic tail, and a Cys-His-rich domain at the carboxyl-terminus [1]. The catalytic domain is the most conserved sequence and the identity is about 35% and the presence of first three domains is common between the five PCSK members (Figure 1). The PCSK family consists of two groups divided by amino acid cleavage sites: typical and atypical (Table 1) [13]. PCSK1, PCSK3, PCSK5, and PCSK6 are typical members and cleave single or paired basic amino acids with the motif (R/K)X_*n*_(R/K)↓, where the arrow indicates the cleavage site and X_*n*_ corresponds to a 0-, 2-, 4-, or 6-amino-acid spacer [2]. PCSK9 is an atypical member and cleaves non-basic residues at the C-terminal end of the motif RX(L/V/I)X↓, where X represents any amino acid except for cysteine and proline [14]. PCSK9 cleaves itself at its internal (V/I/L)FAQ152↓ sequence, and then acts as a binding protein for cell surface receptors [15,16].

**Table 1:** Official and alternative protein name of the nine PCSK members

**Member type**	**Official name**	**Alternative name**
Typical	PCSK1	PCSK1
	PCSK2	PCSK2
	FURIN	PCSK3
	PCSK4	PCSK4
	PCSK5	PCSK5
	PCSK6	PCSK6
	PCSK7	PCSK7
Atypical	MBTPS1*	PCSK8
	PCSK9	PCSK9

•MBTPS1: membrane-bound transcription factor peptidase, site 1.

### PCSKs and lipid metabolism

**Figure 2:**
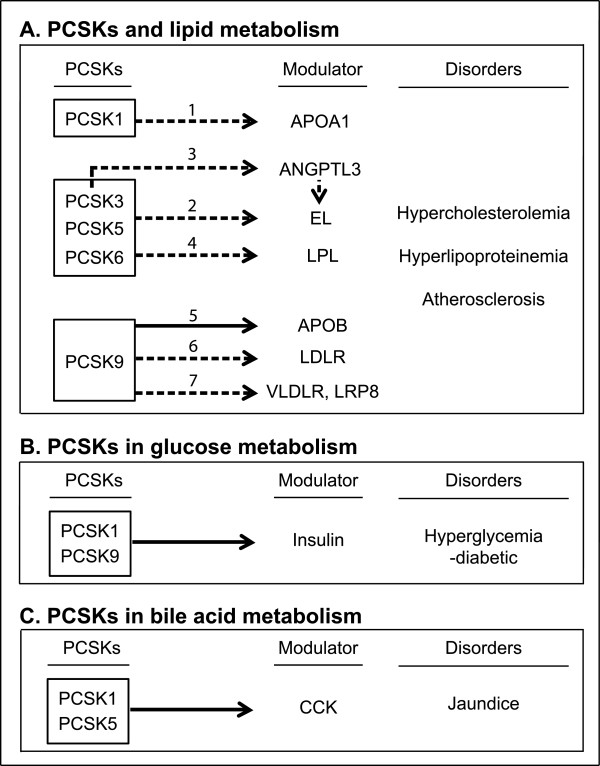
**The role of PCSK members in various metabolic processes and disorders.** The figure illustrates the role of PCSK1, PCSK3, PCSK5, PCSK6, and PCSK9 in lipid **(A)**, glucose **(B)**, and BA **(C)** metabolism. Arrows with 2 different types of lines indicate the relationship between factors: solid lines with arrow indicate a “positive influence” and dotted lines with arrow indicate a “negative influence.” For example, PCSK3, 5, and 6 inactivates EL (Figure 2A(2)). In contrast, solid lines with arrow indicate a “positive influence.” For example, PCSK9 inhibition decreases serum APOB level (Figure 2(5)).

Some PCSK members are involved in regulating the activity of important modulators of lipid metabolism (Figure2A) [3]. The potential role for PCSK1 in lipid metabolism was observed in a study using a loss-of-functional mouse model that carries a mutation in *Pcsk1* (Choi et al., in preparation). The mutation was introduced by treating mice with N-ethyl-N-nitrosourea (ENU), a chemical that induces A- > G transition, which led to an amino acid substitution from asparagine (N) to aspartic acid (D) at position 222. The mutation reduces enzymatic activity of PCSK1 in cleaving downstream substrates [17,18]. In the PCSK1 mutant male mice on a chow-diet for 8 weeks, serum apolipoprotein A1 (APOA1), which is the major component of HDL, was 1.5-2.0 fold increased (Figure 2A(1)), but fasting serum HDL cholesterol concentration in the PCSK1 mutant was similar to wild-type controls. In the PCSK1 mutant mouse livers, APOA1 concentration was ~2.0 fold decreased, while *Apoa1* expression was similar, suggesting that PCSK1 is involved in regulating serum APOA1 concentration and that PCSK1 mutant might increase APOA1 secretion from the liver to serum. It is still unknown why HDL cholesterol concentration is not effected despite the increase in APOA1.

PCSK3, PCSK5, and PCSK6 are involved in lipid metabolism by modulating two members of the TG lipase family. The three PCSK members negatively influences EL activity by cleaving a carboxyl-terminal 18 kDa fragment in EL to suppress the enzymatic activity of EL (Figure 2A(2)) [19]. EL cleavage was induced by transient expression of PCSK3, PCSK5, and PCSK6 in HEK293 cells [20]. PCSK3 may also negatively influence EL activity through angiopoeitin-like protein 3 (ANGPTL3) (Figure 2A(3)). Adenoviral transduction of PCSK3 in hepatocytes led to less cleavage product and increased full-length ANGPTL3 [11]. ANGPTL3 inhibited ~60% of EL activity by transfecting a plasmid expressing murine ANGPTL3 into HEK293 cells stably expressing EL [11]. Lipoprotein lipase (LPL) is another member of the TG lipase family that is similarly inactivated by PCSK-mediated proteolytic cleavage (Figure 2A(4)) [21]. Transient expression of PCSK3, PCSK5, and PCSK6 induced LPL cleavage in HEK293 cells [20].

The first work that linked PCSK9 with lipid metabolism was published by Abifadel and colleagues 10 years ago. Gain-of-function mutations in *PCSK9* were found in familial autosomal dominant hypercholesterolemia patients [22]. The mutations increase LDLR degradation and decrease LDL clearance, which results in an increase in circulating LDL. PCSK9 positively regulates APOB concentration (Figure 2(5)) and inhibition of PCSK9 leads to an increase of LDLR (Figure 2A(6)) and APOB-containing LDL particles are more rapidly cleared. PCSK9 also promotes degradation of the VLDL receptor (VLDLR) and APOE receptor type 2 (LRP8), both of which are important in TG and VLDL metabolism. PCSK9 negatively regulates VLDL concentration through the two receptors (Figure 2A(7)). Ectopic expression of the *PCSK9* gene in HEK293 cells reduced the levels of LRP8 and VLDLR, while PCSK9 inhibition in CHO-1 and HEK293 cell lines increased the levels of VLDLR [23].

### PCSKs and glucose metabolism

PCSK1 and PCSK9 are involved in glucose metabolism (Figure 2B) [1]. PCSK1 and PCSK9 positively regulate pro-insulin conversion to insulin by proteolytic cleavage in the pancreas. A non-synonymous single nucleotide polymorphism in human *PCSK1* (*rs6232*) substitutes asparagine with aspartic acid at codon 221 (N221D). Transfecting recombinant N221D PCSK1 protein in human embryonic kidney 293 cells reduces the proteolytic cleavage of its substrate [24]. The *rs6232* variant is associated with an increase in fasting blood glucose level and a decrease in pancreatic β-cell function and insulin resistance [25]. Insulin was decreased in the pancreas of PCSK9 KO C57BL/6 males, and the males exhibited hyperinsulinemic, hyperglycemic, and glucose-intolerant phenotypes [26].

### PCSKs and BA metabolism

PCSK1 and PCSK5 are involved in BA metabolism (Figure 2C) and positively regulate CCK in the small intestine by converting procholecystokinin (proCCK) to CCK [27]. An immunohistochemical analysis and an *in vitro* cleavage assay showed that PCSK1 co-localizes with proCCK and positively regulates proCCK level. The uncleaved proCCK level was increased and the cleaved CCK level was decreased in the small intestine of mice lacking PCSK1 compared to wild-type control mice [28]. Treating cultured mouse small intestinal cells with small-interfering (si) RNA against *Pcsk5* significantly reduced the secretion of cleaved CCK into the cell culture medium [29]. The secretion of CCK from the small intestine stimulates BA secretion from the gall bladder to the gut. BAs promote lipid absorption and modulate cholesterol levels through enterohepatic circulation. Defects in BA metabolism cause jaundice of the eye and skin.

### PCSKs and HDL metabolism

**Figure 3:**
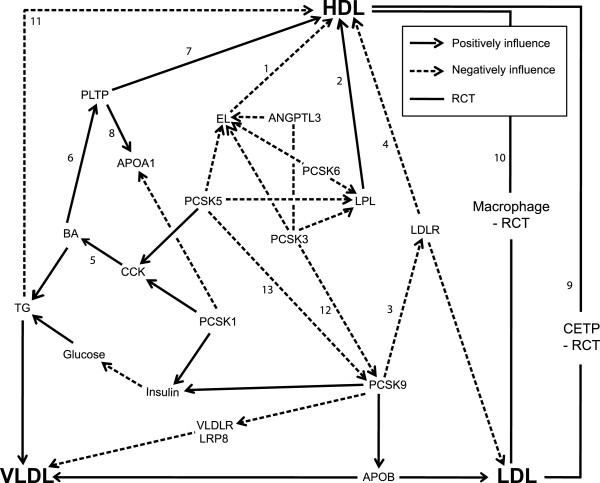
**Integrated physiological model of the molecular and metabolic networking between PCSK members.** The figure illustrates the role of PCSK1, PCSK3, PCSK5, PCSK6, and PCSK9 in the lipoprotein triad (HDL, LDL and VLDL). HDL level and function are synergistically regulated by those metabolic factors: glucose, triglyceride (TG) and BA metabolism. Arrows with 2 different types of lines indicate the relationship between factors: dotted lines with arrow indicate a “negative influence.” For example, LDLR decreases HDL cholesterol concentration (Figure 3(4)). In contrast, solid lines with arrow indicate a “positive influence.” For example, CCK secreted from the small intestine stimulates BA secretion (Figure 3(5)). Solid lines without arrow indicate two major ways of reverse cholesterol transport (RCT). First, cholesteryl ester transfer protein (CETP) exchanges cholesterol in LDL by TG in HDL (Figure 3(9)). Second, HDL takes up cholesterol that is accumulated in macrophage foam cells inside the blood vessel wall (Figure 3(10)). Cholesterol-carrying HDL returns to the liver to recycle or excrete the cholesterol.

PCSK3, PCSK5, and PCSK6 are involved in HDL metabolism by modulating EL and LPL. EL is expressed on the surface of vascular endothelial cells and hydrolyzes phospholipids in HDL. Over-expression of EL in mice leads to a decrease in HDL cholesterol concentration while mice lacking EL show elevated HDL cholesterol concentration (Figure 3(1)) [30]. LPL activation by treating rats with LPL inducing reagent (NO-1886) increased HDL cholesterol concentration [31], showing that LPL activity positively correlates with serum HDL cholesterol concentration (Figure 3(2)). Recent animal studies, including ours, showed that PCSK9 is involved in HDL metabolism. PCSK9 KO male mice on a C57BL/6 background and fed a chow diet exhibited a 30% reduction of HDL cholesterol concentrations [32]. C57BL/6 males fed a high fat diet and then treated with a *Pcsk9* antisense oligonucleotide inhibitor for 6 weeks showed a 54% reduction in HDL cholesterol concentration [33]. In male cynomolgus macaques, treatment with neutralizing antibodies against PCSK9 reduced HDL cholesterol concentrations for the first seven days of treatment [34]. Data from our lab shows the molecular mechanism in which PCSK9-mediated HDL regulation is mainly through low-density lipoprotein (LDLR) [12]. PCSK9 inhibition increases LDLR level, suggesting the negative relationship between PCSK9 and LDLR (Figure 3(3)). Increased LDLR subsequently induces the binding of LDLR to APOE in HDL and thereby leads to rapid HDL clearance. In mice, HDL is the primary cholesterol carrier and the majority of cholesterol is enriched in APOE-containing HDL particles so that reduced level of APOE-containing HDL in the PCSK9 KO mice is likely to be a cause of reduced HDL cholesterol concentration. Thus, LDLR negatively influences HDL cholesterol concentration (Figure 3(4)).

Normal regulation of insulin, glucose, hormones, and lipids is important for HDL homeostasis, which involves synthesis, action, and elimination of HDL lipoproteins [35,36]. Mutations in PCSK1 and PCSK5 may cause abnormal HDL metabolism through mis-regulation of signal molecules in BA. For example, CCK or chenodeoxycholic acid (CDCA) positively influences BA metabolism (Figure 3(5)). When dietary lipids and other nutrients arrive in the gut, cholecystokinin (CCK) secreted from the small intestine stimulates BA secretion from the gall bladder to the gut. The majority of the BA is re-absorbed into the liver via enterohepatic circulation and the reminder is excreted through feces. CDCA is a major component in the BA and functions as a signaling molecule in HDL metabolism. CDCA binds to the farnesoid X-activated receptor (FXR) that regulates lipid and cholesterol metabolism [37]. FXR, a member of the nuclear hormone receptor superfamily, forms a heterodimer with the retinoid X receptor (RXR). The FXR/RXR heterodimer functions as a transcription factor that positively influences phospholipid transfer protein (PLTP) mRNA transcription (Figure 3(6)). Co-transfecting FXR and RXR expression plasmids and CDCA treatment in monkey kidney cells increased PLTP promoter activity. Cholic acid, another major component in the BA, is involved in PLTP regulation [38]. Mice fed a chow diet with cholic acid exhibited increased hepatic PLTP mRNA levels. PLTP is an important factor for HDL conversion and positively influence HDL level (Figure 3(7)). Mice lacking the *Pltp* gene displayed reduced levels of HDL cholesterol (65%) and APOA1 protein (85%) (Figure 3(8)) compared to wild-type control mice [39]. In humans, a non-synonymous mutation of L196W in the *PLTP* gene impairs HDL conversion [40,41].

Maintenance of normal HDL metabolism is critical for normal physiological function of HDL. Reverse cholesterol transport (RCT) is an anti-atherogenic process that occurs between lipoproteins (Figure 3(9)) or between lipoproteins and macrophage foam cells (Figure 3(10)). The efficiency of RCT can be analyzed by measuring the amount of cholesterol in medium and cells. EL deficiency affects efficiency of cholesterol efflux from macrophages. EL knockdown using small hairpin (sh) RNA reduced APOA1-mediated cholesterol efflux [42]. PCSK9 KO decreases the efficiency of cholesterol efflux [12]. Treating PCSK9 KO mouse serum to cultured macrophage foam cells exhibits decreased cholesterol efflux. Thus, abnormal HDL regulation caused by genetic alterations in some PCSK members may interfere with reverse cholesterol transport efficiency.

Normal insulin processing, which influences glucose and triglyceride metabolism, is critical for maintaining normal HDL metabolism [35]. Type II diabetic patients show decreased small HDL particles [43]. Hypertriglyceridemia patients with hyperglycemic phenotype exhibited increased TG-rich HDL with lower stability and shorter plasma residence time compare to cholesterol-rich HDL (Figure 2(11)) [44]. The TG-rich HDL is rapidly catabolized, which subsequently decreases plasma HDL cholesterol concentration [45]. Another potential cause of abnormal HDL metabolism is glycation that leads to conformation changes of proteins. Glycation of amino-acid residues in APOA1 alters its binding affinity to ATP-binding cassette sub-family G member 1 (ABCG1) in the human acute monocytic leukemia cell lines (THP-1). As a result, the ability of cholesterol efflux from THP-1 cells was significantly reduced (< 70%) compared to unglycated APOA1 [46].

## Conclusion and discussion

**Figure 4:**
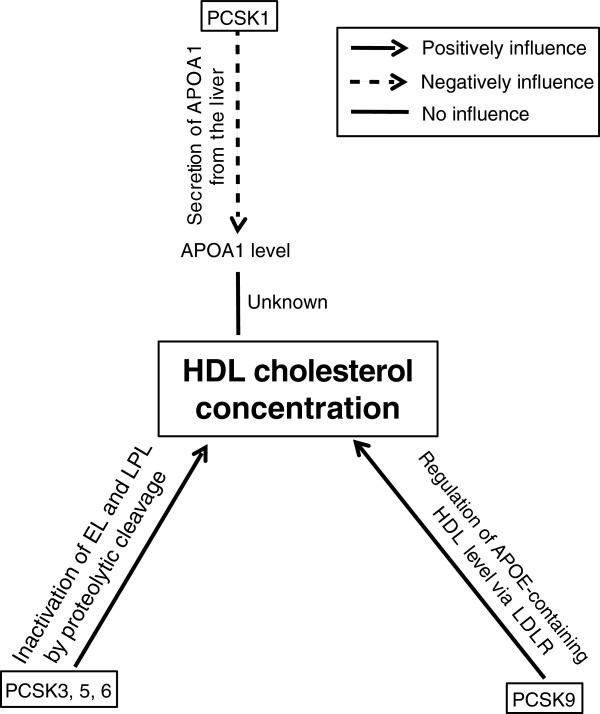
**The role and action of PCSK members in regulating HDL cholesterol concentration.** The figure illustrates the role and action of PCSK1, PCSK3, PCSK5, PCSK6, and PCSK9 in HDL cholesterol concentration. Arrows with 2 different types of lines indicate the relationship between factors: solid lines with arrow indicate a “positive influence” and dotted line with arrow indicates a “negative influence.” Solid line without arrow indicates a “no influence.” PCSK1 negatively influences serum APOA1 level, but does not influence HDL cholesterol concentration, PCSK3, 5 and 6 positively influences HDL cholesterol concentration by modulating enzymatic activities of EL and LPL. PCSK9 positively influences HDL cholesterol concentration by regulating APOE-containing HDL subfraction level through LDLR.

In this review, we summarize how PCSK1, 3, 5, 6, and 9 influence HDL cholesterol concentrations by regulating modulators in various metabolic processes (Figure 4). We also review the current understanding of how five PCSK members are linked not only to abnormal HDL metabolism, but also to other metabolic processes including regulation of lipid, glucose, and BA. To explain the complex relationships between the PCSK members and various metabolic processes, an integrated physiological point of view will be critical to improve our understanding about PCSK members. We propose a model in which PCSK members play pivotal roles in HDL, non-HDL, glucose, insulin, BA, and TG regulation. For example, PCSK1 influences lipoprotein, glucose, and BA metabolism by regulating APOA1, the activity of insulin, and CCK. PCSK3 and PCSK5 influence lipoprotein metabolism by modulating the activity of EL, LPL, and the level of LDLR through PCSK9 cleavage. PCSK9 influences glucose and lipoprotein metabolisms by regulating insulin and LDLR receptor degradation.

Some of the PCSK members have functional redundancy by processing common substrates: insulin by PCSK1 and PCSK9; CCK by PCSK1 and PCSK5; TG lipases (EL and LPL) by PCSK3, PCSK5, and PCSK6. In the latter case, all 3 PCSKs modulate the activity of the TG lipases, but the efficiency of the modulation varies: for EL inactivation PCSK6 has the highest efficiency while PCSK3 has the lowest efficiency; for LPL activation PCSK3 has the highest efficiency while PCSK5 has the lowest efficiency [11]. The PCSK members also regulate one another. The activity of PCSK9 is negatively influenced by two other PCSK members: PCSK3 (Figure 3(12)) and PCSK5 (Figure 3(13)). PCSK3 cleaves PCSK9 at the Arg^218^-Gln^219^ peptide bond in HEK293 cells, and PCSK5 cleaves the same bond, but with lower efficiency compared to PCSK3 [11].

The biology and therapeutic targeting of PCSK members has been recently reviewed [1]. Particularly, therapeutic inhibition of PCSK9 has been proven to be a promising pro-atherogenic LDL cholesterol-lowering treatment. Co-treatment of PCSK inhibitors with drugs that suppress cholesterol synthesis is even more effective in reducing LDL cholesterol in hypercholesterolemia patients [47]. Also, PCSK9 inhibition in the patients did not cause negative impact on anti-atherognic HDL cholesterol concentration in early-phase clinical trial phase I or phase II [47-49]. However, the effect of PCSK9 inhibition on HDL cholesterol concentration is controversial. Some studies used mice showed that PCSK9 inhibition decreases HDL cholesterol concentration [12,32]. Because HDL cholesterol regulation and metabolism differ between mouse and human, we speculate that in human, although the inhibition of PCSK9 affects LDLR level, it might not cause strong enough effect to see significant measurable impact on HDL cholesterol concentration. In humans, cholesterol ester transfer protein (CETP) is a critical factor for regulating HDL cholesterol concentration by transferring cholesterol from HDL to non-HDL particles. In mice lacking CETP, cholesterol is enriched in APOE-containing HDL particles and the particles are cleared through LDLR. In other words, the effect of an increase of LDLR level by PCSK9 inhibition on HDL cholesterol concentration in humans might be smaller relative to its effect in mice. At the same time, studies in nonhuman primates, which have CETP and have similar HDL cholesterol metabolism to humans in comparison to mice, show inconsistent results in regards to the effect of PCSK9 inhibition on HDL cholesterol concentration [34,50]. Treatment with neutralizing antibody against PCSK9 for the first 7 days of treatment decreased HDL cholesterol concentration [34]. Meanwhile, knockdown of PCSK9 by RNAi did not decrease HDL cholesterol concentration [50]. The data from these studies suggest that there are likely to be other factors causing the inconsistent results between studies. First, it is possible that the inconsistent results between studies might be caused by the dosage of PCSK9 inhibition. Knockout of PCSK9 in mice [12,32] decreased HDL cholesterol concentrations and knockdown of PCSK9 by RNAi [50] and overexpression of PCSK9 by adenovirus [51] did not decrease HDL cholesterol concentrations. Second, it is also possible that whether or not PCSK9 targeting directs liver might cause the inconsistent results. Liver-specific siRNA silencing [50] and overexpression of PCSK9 by adenovirus, which is known to primarily target the liver, do not decrease HDL cholesterol concentration. PCSK9 in other tissues remains unaffected and might contribute to the lack of decreased HDL cholesterol concentration. The no change in HDL cholesterol concentration despite the change in LDLR by PCSK9 inhibition [50] and overexpression [51] remains unclear and additional studies would be needed to address this observation. If the method is indeed the cause of the variation in HDL cholesterol, then additional studies may provide valuable information about the effectiveness of different methods. At the same time, the use of neutralizing antibodies against PCSK9 in several clinical trials [47-49] show no negative impact on HDL cholesterol concentration. Considering the importance of PCSK9 as a promising therapeutic target, it will be important to understand whether the inconsistent results are due to different dosage (knockout versus knockdown), CETP (presence vs absence), or target (gene vs protein; liver vs organism). The molecular and physiological functions of PCSK members in HDL metabolism are likely more complex than we currently know. In particular, our understanding of compensatory mechanisms between PCSK members is also limited. We look forward to continued studies that will improve our understanding and allow more precise pharmacological regulation of PCSK for the treatment of metabolic disease.

## Competing interests

The authors declare that they have no competing interests.

## Authors’ contributions

SC drafted the manuscript; RK revised the manuscript and gave the final approval of the version to be published. Both authors read and approved the final manuscript.

## Authors’ information

SC: Ph.D. candidate in the cooperative pre-doctoral program at The Jackson Laboratory, Bar Harbor, ME 40609, USA. RK: assistant professor at the Jackson Laboratory and adjunct research scientist at The Mount Desert Island Biological Laboratory, Bar Harbor, ME 04609, USA.
